# Controlling Oxygen Vacancies in BiFeO_3_ Thin Films via Pyrolysis Temperature and O_2_ Annealing

**DOI:** 10.3390/nano16070395

**Published:** 2026-03-25

**Authors:** Saulo P. Reis, Marco Antonio M. Teixeira, Fernando B. Minussi, Maria Jesus Hortigüela, Gonzalo Otero-Irurueta, Leandro Bufaiçal, Eudes B. Araújo

**Affiliations:** 1 Federal Institute of Education, Science and Technology of São Paulo, Votuporanga 15503-110, Brazil; spdreis@ifsp.edu.br; 2 Department of Physics and Chemistry, São Paulo State University, Ilha Solteira 15385-000, Brazil; marco.teixeira@unesp.br; 3 Department of Physics, Federal University of São Carlos, São Carlos 13565-905, Brazil; 4 TEMA—Centre for Mechanical Technology and Automation, University of Aveiro, 3810-193 Aveiro, Portugal; mhortiguela@ua.pt (M.J.H.); otero.gonzalo@ua.pt (G.O.-I.); 5 Instituto de Física, Universidade Federal de Goiás, Goiânia 74001-970, Brazil; lbufaical@ufg.br

**Keywords:** bismuth ferrite, thin films, oxygen vacancies, point defects

## Abstract

Bismuth ferrite (BiFeO_3_) is a promising material for developing the next generation of multifunctional electronic devices. However, the production of high-quality BiFeO_3_ thin films is compromised by the tendency for structural and electronic defects to form during synthesis, which degrades their functional properties. In this work, BiFeO_3_ thin films were prepared by chemical solution deposition to determine optimal conditions for minimizing oxygen vacancies and to evaluate the impact of these point defects on their physical properties. The films were pyrolyzed at 300 °C for 60 min and 360 °C for 10 min, and crystallized in air and in an O_2_ atmosphere, at 600 °C and 640 °C for 40 min. High oxygen vacancies were observed in films prepared at low pyrolysis temperatures and crystallized in air, whereas oxygen vacancies were minimized in the film pyrolyzed and crystallized at high temperatures in an O_2_ atmosphere. The oxygen vacancies markedly affected the films’ physical properties, leading to increased dielectric loss, dielectric dispersion, dc conductivity, and leakage current, with consequent degradation of photovoltaic and magnetic performance. These findings highlight the critical importance of controlling synthesis parameters to suppress oxygen vacancy formation and achieve high-quality BiFeO_3_ thin films.

## 1. Introduction

Bismuth ferrite (BiFeO_3_) is a prototypical multiferroic material characterized by a coexistence of the antiferromagnetic (T_N_~643 K) and ferroelectric (T_C_ = 1103 K) phases at room temperature [1,2]. Due to its large spontaneous polarization and the presence of magnetoelectric coupling at room temperature, the BiFeO_3_ has attracted considerable attention as a promising material with potential to develop the next generation of multifunctional electronic devices [3,4]. Controlling magnetization with electric fields and vice versa has been a frequent motivation for research into this multiferroic, with its potential for energy-efficient, high-density, and fast information storage, as well as for new concepts in sensors and logic devices [5,6]. Furthermore, exploring the synthesis of BiFeO_3_ thin films and their physical properties is highly relevant, as nanoscale engineering is essential for integrating high-tech microelectronics and nanoelectronics. However, despite the large number of scientific articles published in recent years on BiFeO_3_ thin films, their effective practical technological application is not yet a reality due to several critical problems that remain unsolved.

The technological application of materials requires producing high-quality materials with rigorous control over their mechanical, physical, and chemical properties, which are necessary for effective use. However, the practical realization of high-quality BiFeO_3_ thin films is strongly hindered by the tendency to form structural and electronic defects during growth, which degrade their functional properties by increasing leakage currents [7], leading to phase instability and reduced ferroelectric performance. Most physical property degradation is often associated with point or complex defects related to stoichiometry fluctuations [8,9], whose mechanisms include valence fluctuations of the Fe ions [10], the formation of oxygen vacancies [11], and inhomogeneities associated with the Bi volatilization during synthesis [12]. Bismuth, iron, and oxygen vacancies are the primary defects in the BiFeO_3_, with Bi and Fe assuming cation vacancies for charge states −3, −2, −1, 0, while oxygen vacancies appear fully ionized ($V_{O}^{\cdot \cdot }$), partially ionized ($V_{O}^{\cdot }$) or neutral ($V_{O}^{x}$) [13]. In addition, free holes and free electrons can be produced by ionized defects [14]. Bi vacancies introduce three holes in the system, while the ionization of oxygen vacancies justifies the presence of free electrons. Theoretical studies demonstrate that Bi and Fe vacancies are dominant defects in oxidizing conditions. In contrast, Bi and O vacancies are dominant under reducing conditions, leading to p-type or n-type conductivity, respectively [15]. The presence of oxygen vacancies leads to the formation of Fe^2+^ ions, such that both can contribute to the high leakage current. For these reasons, manipulating oxygen vacancies by Fe site substitution [16] or by doping the Bi site [17,18] has been used as an alternative to reduce leakage current and improve photovoltaic response in BiFeO_3_ thin films [19]. Due to these complex defects, it is not surprising that different physical properties are often reported in the literature for the BiFeO_3_.

The quality of BiFeO_3_ thin films prepared by Chemical Solution Deposition (CSD) method via spin coating is strongly governed by the nucleation and crystallization mechanisms that occur during thermal processing [20,21]. Defect formation, phase instability, and electrical leakage are not merely consequences of processing conditions but are intrinsically linked to the kinetics and thermodynamics of perovskite phase nucleation [22] and to subsequent grain growth related to the synthesis method. Focusing attention on chemically deposited methods, nucleation and crystallization processes are closely coupled to defect chemistry, particularly oxygen vacancy formation [21,23]. Nucleation of the perovskite phase typically occurs after solvent evaporation and organic decomposition during a temperature-annealing step, often called pyrolysis, although the heat treatment occurs in the presence of oxygen [24,25]. From a thermodynamic perspective, the formation of the perovskite phase competes with secondary phases that often possess lower nucleation barriers, particularly under Bi-deficient or kinetically limited conditions. Also, bismuth’s high volatility increases the critical nucleation energy of the BiFeO_3_ phase, favoring the formation of iron-rich parasitic phases. Introducing excess bismuth into the precursor solution is a strategy that effectively stabilizes the chemical potential of Bi and lowers the nucleation barrier for the perovskite phase [26]. Regarding crystallization kinetics, high annealing temperatures enhance grain growth but exacerbate bismuth volatilization and oxygen vacancy formation, while insufficient oxygen incorporation during crystallization leads to charge imbalance and mixed Fe^2+^/Fe^3+^ states, increasing electronic conductivity. As an alternative, annealing in oxygen-rich atmospheres and post-deposition oxygen treatments are often used to stabilize the Fe^3+^ state during grain growth, thereby reducing oxygen vacancy concentration. In summary, understanding the origin of these defects, their consequent effects on structural, dielectric, and electrical properties, and their effective control are fundamental challenges for promoting the transition from academic studies to the announced practical technological applications. Given that context, it is clear that controlling defects in bismuth ferrite-based materials is critical to their application. While this has been an enduring area of interest, most studies focus on the manipulation of defects and properties through doping [27,28,29], whereas only a few works [30,31] have addressed the crucial role of the first thin-film synthesis steps in shaping defects and their consequent properties. Still, even though some studies on this topic have been published, a thorough connection between the synthesis, structure, and multiple functional properties of BiFeO_3_ films remains missing.

To address the current literature on this relevant topic, we herein address the effects of processing on the control of oxygen vacancies in BiFeO_3_ thin films prepared by the CSD method and the consequent impact on their structural, dielectric, electrical, optical, photovoltaic, and magnetic properties. For this purpose, we produced three BiFeO_3_ thin films under different preparation conditions to determine how oxygen vacancies are linked to their crystallization kinetics and physical properties. Two films were prepared in an open-atmosphere electric furnace under the same pyrolysis and crystallization conditions. Nucleation at low-temperature pyrolysis induces oxygen vacancies in both films, but one of them was subjected to a long period of subsequent post-annealing in an O_2_ atmosphere to correct the imbalance. A third film was prepared under an O_2_ atmosphere at higher pyrolysis and crystallization temperatures. The main objective of the study is to make clear that once oxygen vacancies are established during synthesis, there are practically no alternatives to correct the imbalance in subsequent steps, reinforcing the importance of controlling point defects during nucleation and crystallization.

The study aims to elucidate how the oxygen atmosphere and pyrolysis temperature influence the structural evolution of BiFeO_3_ thin films and their physical properties. The novelty of the present investigation lies in the variation in pyrolysis temperature and oxygen environment during crystallization and post-annealing of spin-coated BiFeO_3_ thin films, followed by a comparative analysis of their effects on physical properties. Rather than focusing on the material itself, the study emphasizes how different thermal treatments in oxygen and air environments affect the microstructural development and overall film quality of BiFeO_3_ thin films.

## 2. Materials and Methods

The BiFeO_3_ thin films studied in the present work were prepared from an acetic acid chemical solution route using Bi(NO_3_)_3_·5H_2_O (Sigma-Aldrich, St. Louis, MO, USA, 99.9%) and Fe(NO_3_)_3_.9H_2_O (Sigma-Aldrich, 99.9%) as starting reagents. Dissolving the appropriate amount of reagent into a solution containing 1 mL of 2-methoxyethanol (Sigma-Aldrich, 99.9%) and 5 mL of glacial acetic acid (Sigma-Aldrich, 99.9%) at 50 °C under stirring for 10 min yielded a chemical solution. After complete homogenization, the solution temperature was raised to 80 °C, under stirring for 30 min, and then the heating was switched off. Finally, 3 mL of glacial acetic acid was added to the room temperature to obtain a final 0.16 M solution. Table 1 summarizes the conditions used for the preparation of the studied BiFeO_3_ thin films.

For film preparation, the chemical solution was initially deposited onto Pt/TiO_2_/SiO_2_/Si(100) substrates by spin coating at 5000 rpm for 30 s, followed by thermal annealing in an open electric furnace (open atmosphere) for the pyrolysis (to remove water and organics) at 300 °C for 60 min for the BFO1 and BFO2 films, and at 360 °C for 60 min for the BFO3 film. For each film, the second and subsequent layers were sequentially deposited on the previously pyrolyzed film following the same protocol to increase the film thickness. After pyrolysis and completing eight depositions, the BFO1 film was crystallized in air (open atmosphere) at 600 °C for 40 min, the BFO2 was also crystallized in air at 600 °C for 40 min, and after completing the crystallization, it was post-annealed at 600 °C for 5 h under an O_2_ atmosphere at 18.5 psi. Finally, the BFO3 film was crystallized in an O_2_ atmosphere at 18.5 psi at 640 °C for 40 min, without additional post-annealing. The final films had an average thickness of ~400 nm.

Energy-dispersive X-ray spectroscopy (EDS) attached to a scanning electron microscope (Carl Zeiss EVO LS15; Carl Zeiss, Oberkochen, Germany) was used to analyze the elemental composition of the BiFeO_3_ thin films. The analyses were conducted at 30 kV on samples coated with a thin gold film. The quantification was performed by statistical analysis of images from six different regions in each sample. The mean deviation (δ) was a statistical measure used to calculate the average deviation from the mean value of the EDS data set. Thus, the obtained quantification, along with the mean deviation, provides reliability for interpreting EDS results.

The crystalline structure of the films was characterized by X-ray diffraction (XRD) using a Rigaku Ultima IV diffractometer (Rigaku, Tokyo, Japan) with CuKα radiation (λ = 1.5406 Å) at room temperature, under 40 kV and 40 mA at the 20° ≤ 2θ ≤ 60° range. Rietveld refinements of the XRD data were carried out using GSAS-II software and EXPGUI interface. Pseudo-Voigt functions were used to define the peak profiles for all refinements, while a sixth-order polynomial was used to describe the background. The occupancy parameters of the atoms were fixed at the nominal composition, but the scale factor, zero correction, background, half-width parameters, lattice parameters, positional coordinates, and isotropic thermal parameters were refined. In addition, Raman spectroscopy was also used to study the short-range structure. Raman measurements were performed using a confocal Raman BX51 Voyage TM (Perkin Elmer, Shelton, CT, USA) with 785 nm laser excitation and spectral resolution of 3 cm^−1^.

X-ray photoelectron spectroscopy (XPS) was used to study the chemical properties and valence states of the BFO films. The XPS spectra were acquired in an Ultra High Vacuum (UHV) system with a base pressure of 2 × 10^−10^ mbar. The system is equipped with a hemispherical electron energy analyzer (SPECS Phoibos 150; SPECS Group, Berlin, Germany), a delay-line detector, and a monochromatic AlKα (1486.74 eV) X-ray source. High-resolution spectra were recorded at a normal emission take-off angle and with a pass energy of 35 eV, which provides an overall instrumental peak broadening of 0.65 eV. XPS calibration was performed using 284.8 eV as the reference for adventitious carbon. Charge compensation was done by using a low-energy electron flow gun.

For the electrical measurements, gold electrodes of 300 μm in diameter were sputtered onto the films using a shadow mask to form metal/ferroelectric/metal capacitors. An Agilent 4284A LCR meter (Agilent, Santa Clara, CA, USA) was used to measure the complex dielectric permittivity ($\varepsilon^{*}=\varepsilon^{\prime }+i\varepsilon^{\prime \prime }$), impedance ($Z^{*}=Z^{\prime }-iZ^{\prime \prime }$), the electric modulus $M^{*}=1/\varepsilon^{*}=M^{\prime }-iM^{\prime \prime }$, and the ac electric conductivity (σ) at different frequencies (10^2^–10^6^ Hz) and temperatures (300–500 K). For the analysis of impedance spectroscopy data, an equivalent circuit composed of a series combination of RC and RC-CPE components was considered [32,33], representing the effects of grains and grain boundaries, respectively. The introduced constant phase element (CPE) indicates a departure from the ideal Debye model. From the model, the following equations for the real and imaginary parts of impedance and electric modulus were used to fit the experimental data as a function of the angular frequency (ω=2πf):

(1) $$Z^{\prime }=\frac{R_{gb}\left[1+R_{gb}A\omega^{n}cos\left(\frac{\pi n}{2}\right)\right]}{{\left[1+R_{gb}A\omega^{n}cos\left(\frac{\pi n}{2}\right)\right]}^{2}+{R_{gb}\left[\omega C_{gb}+A\omega^{n}sin\left(\frac{\pi n}{2}\right)\right]}^{2}}+\frac{R_{g}}{1+{\left(\omega {R_{g}C}_{g}\right)}^{2}}$$

(2) $$Z^{\prime \prime }=\frac{R_{gb}^{2}\left[\omega C_{gb}+A\omega^{n}sin\left(\frac{\pi n}{2}\right)\right]}{{\left[1+R_{gb}A\omega^{n}cos\left(\frac{\pi n}{2}\right)\right]}^{2}+{R_{gb}\left[\omega C_{gb}+A\omega^{n}sin\left(\frac{\pi n}{2}\right)\right]}^{2}}+\frac{\omega R_{g}C_{g}}{1+{\left(\omega R_{g}C_{g}\right)}^{2}}$$

(3) $$M^{\prime }=\left[\frac{\omega C_{0}R_{gb}^{2}\left[\omega C_{gb}+A\omega^{n}sin\left(\frac{\pi n}{2}\right)\right]}{{\left[1+R_{gb}A\omega^{n}cos\left(\frac{\pi n}{2}\right)\right]}^{2}+{R_{gb}\left[\omega C_{gb}+A\omega^{n}sin\left(\frac{\pi n}{2}\right)\right]}^{2}}+\frac{\omega R_{g}C_{g}}{1+{\left(\omega R_{g}C_{g}\right)}^{2}}\right]\;$$

(4) $$M^{\prime \prime }=\left[\frac{\omega C_{0}R_{gb}\left[1+R_{gb}A\omega^{n}cos\left(\frac{\pi n}{2}\right)\right]}{{\left[1+R_{gb}A\omega^{n}cos\left(\frac{\pi n}{2}\right)\right]}^{2}+{R_{gb}\left[\omega C_{gb}+A\omega^{n}sin\left(\frac{\pi n}{2}\right)\right]}^{2}}+\frac{R_{g}}{1+{\left(\omega {R_{g}C}_{g}\right)}^{2}}\right]$$

where $C_{0}=\varepsilon_{0}a/d$ (d is the sample thickness and a the electrode area); $R_{g}$ is the grain resistance; $C_{g}$ is the grain capacitance; $R_{gb}$ is the grain boundary resistance; and $C_{gb}$ is the grain boundary capacitance.

The leakage current-voltage (I−V) and photovoltaic characteristics of BiFeO_3_ films were characterized using a Keithley 6517B electrometer (Tektronix, Beaverton, OR, USA). The photovoltaic responses J(t) were obtained by illuminating the films between the electrodes with a monochromatic green laser λ = 532 nm (hν = 2.33 eV). Magnetic measurements were performed using a Quantum Design PPMS-VSM magnetometer (Quantum Design, San Diego, CA, USA) at a H sweep rate of 30 Oe/s. The magnetization as a function of temperature was measured under zero-field cooling (ZFC) and field cooling (FC) regimes in the temperature range 5–390 K, and magnetization hysteresis loops were measured at 5 K and 300 K with an applied magnetic field of ±30 kOe.

## 3. Results and Discussion

**Figure 1:**
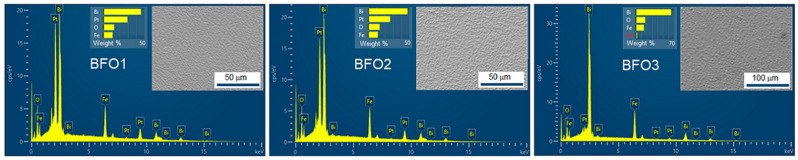
EDS spectra and scanning electron micrographs of the analyzed areas of the studied BiFeO_3_ thin films.

The prepared BiFeO_3_ films for the present study had homogeneous, smooth, and crack-free surfaces. Atomic elemental analysis by EDS quantifies stoichiometry across eight different surface areas and confirms the nominal composition of the synthesized BiFeO_3_ films within experimental accuracy. Figure 1 shows the EDS spectra and corresponding scanning electron micrograph images used for statistical analysis and quantification. The atomic percentages of the elements from the statistical analysis and the corresponding Bi/Fe, O/Bi, and O/Fe molar ratios were summarized in Table 2.

### 3.1. Structural Analysis

**Figure 2:**
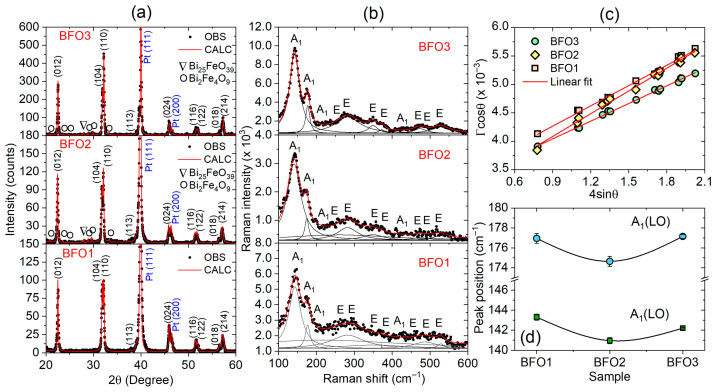
(**a**) Observed (points) and calculated (red lines) XRD patterns of the studied BiFeO_3_ films (BFO1, BFO2, and BFO3). Traces of the secondary Bi_25_FeO_39_ (∇) and Bi_2_Fe_4_O_9_ (O) phases are observed. (**b**) Raman spectra of the studied BiFeO3 thin films at room temperature. Black points are the experimental data; full fitting (red line) and Lorentzian curves (black lines) were used to fit and represent the individual Raman peaks corresponding to different phonon modes. (**c**) Williamson–Hall plots and (**d**) Raman shifts for the two A1 (LO) modes of the BFO1, BFO2, and BFO3 films.

The long-range crystalline structure of the studied films was characterized by X-ray diffraction, and Raman microscopy complements the short-range order structural analysis. Figure 2a,b shows the XRD patterns and Raman spectra of the studied BiFeO_3_ thin films. In Figure 2a, no preferred orientations were observed for all XRD patterns, and the indexed (*hkl*) peaks refer to the rhombohedral structure of the BiFeO_3_ with an R3c space group. The peaks Pt (111) and Pt (200) refer to the cubic structure (*a* = 3.892 Å) of the platinum substrate with an $Fm\bar{3}m$ space group. A single-phase BiFeO_3_ is observed in Figure 2a in the BFO1 film. On the other hand, traces of the secondary Bi_25_FeO_39_ (sillenite-type structure) and Bi_2_Fe_4_O_9_ (mullite-type structure) phases were observed in coexistence with the BiFeO_3_ phase in the BFO2 and BFO3 films. For the BFO1 film, the Rietveld refinement was performed assuming a single BiFeO_3_ phase with a rhombohedral structure (space group *R3c*). For the BFO2 and BFO3 films, the refinements assumed a coexistence model of the rhombohedral (space group *R3c*), cubic (space group *I23*), and orthorhombic (space group *Pbam*) structures, respectively associated with the BiFeO_3_, Bi_25_FeO_39_, and Bi_2_Fe_4_O_9_ phases. In addition, the cubic structure of the platinum (space group $Fm\bar{3}m$) was also included in all refinements. Based on refinements, the amounts of both Bi_25_FeO_39_ and Bi_2_Fe_4_O_9_ phases in BFO2 and BFO3 films were estimated to be less than 5%, given the fitting accuracy. The parameters obtained from refinements are summarized in Table 3.

The lattice parameters and cell volume obtained for all samples are very similar and in good agreement with those reported for BiFeO_3_ single crystals [34]. From a structural perspective, no differences were observed in the studied films due to variations in processing under different annealing atmospheres. On the other hand, the different crystallization temperatures strongly influence the intensity of the XRD peaks. The intensity of the XRD peaks is associated with the number of atoms in the crystal contributing to the diffraction, such that higher peak intensities indicate a higher degree of crystallinity. The more intense X-ray diffraction peaks indicate higher crystallinity for the BFO3 film, while the lower peak intensities indicate moderate crystallinity for BFO2 and lower crystallinity for the BFO1 sample. As BFO2 and BFO1 films were prepared at the same crystallization temperature (600 °C) under different atmospheres, the very similar XRD peak intensities indicate that the annealing atmosphere has no pronounced effects on the crystallinity. In contrast, the increase in crystallization temperature (640 °C) of the BFO3 film leads to higher crystallinity, indicating that temperature is more effective in improving the film crystallinity. In addition, the slope of the plot drawn between Γcosθ versus 4sinθ from the Williamson–Hall approach in Figure 2c gives the values of microstrain, while the y-intercept gives the values of crystallite size. For the studied BFO1, BFO2, and BFO3 films, the obtained crystallite sizes were 43, 48, and 45 nm, while the microstrains were 1.2 × 10^−3^, 1.3 × 10^−3^, and 1.1 × 10^−3^, respectively. Despite the expected link between synthesis and processing conditions, the crystallite sizes and microstrains are the same across the films.

Figure 2b shows the Raman spectra of the three studied BiFeO3 films, recorded at room temperature in the range of 100–600 cm^−1^. According to the group theory, BiFeO3 with rhombohedral R3c structure exhibits 13 (4A1 + 9E) Raman-active modes, which were fitted in Figure 2b using Lorentzian curves for each mode. The assignment for the Raman modes of the studied BiFeO_3_ films is summarized in Table 4 and compared with results from the literature for single crystals [35] and epitaxial BiFeO_3_ thin films [36]. The peaks at 141, 174, 220, and 430 cm^−1^ are A1-symmetry longitudinal-optical A1 (LO) phonons, while the peaks at 260, 281, 349, 371, 469, 480, and 527 cm^−1^ are all related to the transverse-optical E (TO) modes, considering as a reference the Raman spectrum of the BFO3 film. Similarly to the observed in the XRD patterns in Figure 2a, the higher Raman intensity peaks in Figure 2b of the BFO3 film, which was crystallized at a higher temperature (640 °C), compared to the BFO1 and BFO2 films, confirm its higher crystallinity. On the other hand, the increase or decrease in Raman shift provides a powerful tool for probing property changes directly affected by the synthesis conditions, since Raman frequency shift is susceptible to internal and external factors associated with structural and physical properties, such as crystallite size and crystallinity [37], phase composition and oxygen vacancies [38], defects and strains [39], and compressive/tensile stresses [40]. The Raman shift in the A1 (LO) modes in the BFO2 film shifts to lower frequencies or longer wavelengths (red shift), in comparison to the BFO1 film modes around 144 cm^−1^ and 177 cm^−1^, as shown in Figure 2d. Despite the red shift often being attributed to improved crystallinity in the literature, this does not appear to be the case, as BFO1 and BFO2 exhibit similar crystallinity, whereas BFO3 shows higher crystallinity. The redshift in the BFO2 film indicates a direct effect of the synthesis conditions, possibly associated not only with oxygen vacancies, but also with other defects that could not be explicitly identified.

### 3.2. X-Ray Photoelectron Spectroscopy Analysis

**Figure 3:**
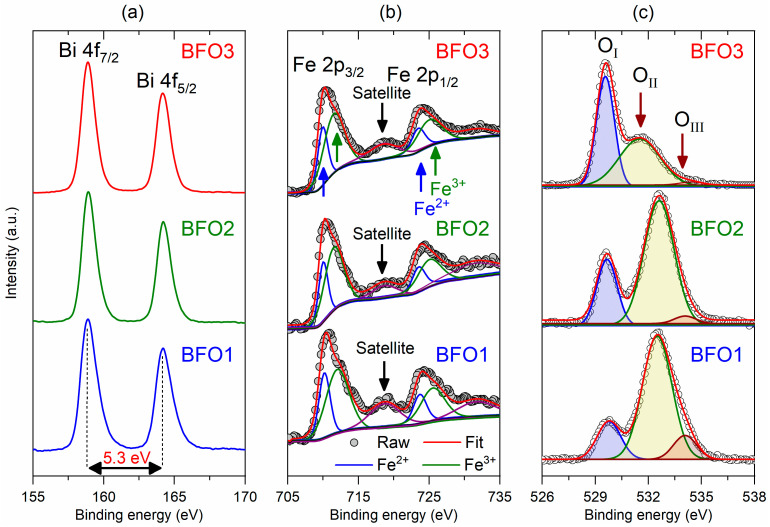
X-ray photoelectron spectroscopy (XPS) spectra of (**a**) Bi 4f, (**b**) Fe 2p, and (**c**) O 1s for the studied BFO1, BFO2, and BFO3 thin films. Open circles in (**b**,**c**) are experimental data, while red lines are the fits.

XPS measurements were conducted to determine the oxidation states, chemical shifts, and oxygen vacancies in the studied BiFeO3 films. Figure 3 shows the XPS spectra of the (a) Bi 4f, (b) Fe 2p, and (c) O 1s core levels for the studied BiFeO_3_ thin films. Figure 3a shows that the Bi 4f core levels of all samples are also similar, such that Bi 4f_7/2_ and Bi 4f_5/2_ were centered at BEs of 158.9 eV and 164.2 eV, with a spin–orbit splitting of 5.3 eV. These results were ascribed to Bi^3+^ and agreed with reports in the literature for BiFeO_3_ thin films [41]. In Figure 3b, the Fe 2p regions were fitted by using a Shirley background and Lorentzian functions. For all samples, the Fe 2p core levels were quite similar. They observed two main peaks ascribed to Fe 2p_3/2_ and Fe 2p_1/2_, with a spin–orbit splitting of 13.6 eV. Furthermore, the results in the present work reveal the coexistence of Fe^2+^ and Fe^3+^ ions in the studied BiFeO_3_ films. Moreover, a satellite peak related to Fe^2+^ and Fe^3+^ ions clearly appeared in the spectra at a binding energy (BE) of about 718.6 eV [42], as indicated in Figure 3b. This satellite peak is characteristic of oxidized Fe atoms. On the other hand, the main peaks could be fitted with two components corresponding to Fe^3+^ and Fe^2+^ [43,44] centered at BEs of about 711.5 eV and 709.9 eV, respectively. While Fe^3+^ was ascribed to the Fe atoms in the pristine BiFeO_3_ samples, Fe^2+^ derives from the surface defects, i.e., oxygen vacancies [40].

Despite reports in the literature stating that the satellite peak for Fe 2p_3/2_ at a difference of 8 eV indicates only the Fe^3+^ presence, the asymmetries observed in Figure 3b for both Fe 2p_3/2_ and 2p_1/2_ peaks clearly suggest a coexistence of Fe^2+^ and Fe^3+^ in the present case. In this case, the satellite peak will also be a combination of Fe^2+^ and Fe^3+^ effects. The obtained Fe^3+^/Fe^2+^ = 2.1 ratios between the oxidation states of Fe were essentially the same for all films. Therefore, Fe^2+^ and Fe^3+^ ions coexist in the studied BiFeO_3_ thin films, but Fe^3+^ ions are predominant compared to Fe^2+^ ions. The presence of Fe^2+^ ions is indicative of the existence of oxygen vacancies, since $V_{O}^{\cdot }\to V_{O}^{\cdot \cdot }+e^{\prime }$ leads to ${Fe}_{Fe}^{x}+e^{\prime }\to {Fe}_{Fe}^{\prime }$ [41]. However, it is worth noting that charge compensation can also occur via non-iron defect centers. The oxygen vacancies detected by O 1s can also be compensated by electronic charge trapping or surface defect states rather than by a one-to-one conversion of Fe^3+^ to Fe^2+^, keeping the Fe^3+^/Fe^2+^ ratio relatively constant across different film preparation conditions, which can justify the contrast between the nearly constant Fe^3+^/Fe^2+^ ratio for all films compared to the large differences in oxygen vacancy concentration observed in the different samples from the O 1s analysis.

The O 1s core levels exhibit three peaks labeled O_I_, O_II_, and O_III_ in Figure 3c, which are attributed to the oxygen-metal bond, the dangling bond, and surface-adsorbed oxygen, respectively, as previously reported for BiFeO_3_ thin films [41]. The O_I_ peak at ~529.6 eV remains constant across all studied BiFeO_3_ films. The O_II_ peak appeared at binding energies ~532.3 eV in BFO1 and BFO2 films, and ~531.7 eV in BFO3 film, while the O_III_ peak appeared at ~534.0 eV in BFO1 and BFO2 films and is not present in BFO3 film, pyrolyzed at 360 °C and crystallized in O_2_ atmosphere at 640 °C. The O_II_ peaks are higher than O_I_ peaks for both BFO1 and BFO2 films, while the O_I_ peak is higher than O_II_ peak in the BFO3 film, indicating a strong influence of the processing on the oxygen vacancies and adsorbed oxygen in the final films. In addition, the asymmetry observed in Figure 3c at O_II_ peak in XPS spectra of BFO1 film, pyrolyzed at 300 °C and crystallized in open atmosphere (in air) at 600 °C, suggests the coexistence of oxygen vacancies with different oxidation states ($V_{O}^{\cdot }$, $V_{O}^{\cdot \cdot }$). The O_III_ is ascribed to oxygen vacancies with higher oxidation states ($V_{O}^{\cdot \cdot }$) as plausible defects in the BiFeO_3_ system [14]. So, the XPS spectra of BFO films were fitted using three Lorentzian functions to simulate the presence of $V_{O}^{\cdot }$ and $V_{O}^{\cdot \cdot }$ oxygen vacancies, in addition to the lattice oxygen. Previous works indicated that O_II_ is mainly associated with hydroxyl groups occupying oxygen defects in BiFeO_3_ films [41,42], but is also associated with oxygen contamination, including oxidized organic groups and adsorbed H_2_O [45]. In our case, although post-annealing in an O_2_ atmosphere potentially reduces the oxygen vacancies compared to the film crystallized at the same temperature (600 °C) in an open atmosphere, the pyrolysis at low temperature (300 °C) probably plays a more prominent role in introducing defects associated with incomplete organic removal during the layer-by-layer film deposition. It is important to mention that bismuth volatilization is widely reported to occur in Bi-containing oxide materials, leading to vacancies and stoichiometric deviations. Even though we do not discard the possibility that it may happen, the fact that bismuth-related XPS data are the same for the three films and that XRD reveals that no Fe-rich phases (e.g., Bi_2_Fe_4_O_9_ and Fe_2_O_3_) are formed alone strongly suggests that bismuth defects should not be the main reason for markedly different properties, as shown in the latter.

The oxygen vacancy content can be estimated from the relative peak’s intensity ratio (RIR) relation $RIR=I_{OM}/\left(I_{DL}+I_{ADS}\right)$, such that $I_{OM}$, $I_{DL}$ and $I_{ADS}$ are the relative peak intensities of the oxygen-metal bond, dangling bond, and adsorbed oxygen, respectively [46]. Thus, a lower RIR value indicates a higher concentration of oxygen vacancies. The calculated RIR values of the BFO1, BFO2, and BFO3 films, from fits in Figure 3c, were 0.30, 0.55, and 2.26, respectively, indicating that the concentration of oxygen vacancies is higher in the BFO1 film and lower in the BFO3 film, while the BFO2 film shows an intermediate concentration of oxygen vacancies. The RIR values indicate that the total oxygen vacancy ($V_{O}=V_{O}^{\cdot }+V_{O}^{\cdot \cdot }$) is smaller in the film post-annealed in an O_2_ atmosphere (BFO2) than in the film crystallized in an open atmosphere (BFO1), both of which were crystallized at the same temperature (600 °C). These results indicate that the decrease in oxygen vacancies in BFO2, compared to the BFO1 film, lowers the O_III_ oxygen density, suggesting that post-annealing in an oxygen atmosphere reduces surface-adsorbed oxygen. Also, the oxygen vacancies are not neutral in these films but fully ionized ($V_{O}^{\cdot \cdot }$) or partially ionized ($V_{O}^{\cdot }$). Furthermore, increasing the pyrolysis temperature to 360 °C for 10 min and then crystallizing at 640 °C for 40 min under an O_2_ atmosphere significantly reduces oxygen vacancies in the BFO3 film, indicating the importance of establishing ideal conditions for defect control during synthesis.

In summary, given the discussion above, it is important to note that the XPS technique is limited by its extreme surface sensitivity (typically 10 nm from the surface), so that bulk properties are not accessible. Although we did not use complementary techniques to assess surface adsorption or contamination in the studied thin films, the standard analyses indicate that the XPS data obtained in the present work reflect the surface chemical composition rather than the entire film. Therefore, justifying that the O 1s-based RIR in XPS reflects the bulk oxygen vacancy concentration rather than surface contamination requires a combination of meticulous sample preparation, careful peak fitting, and validation via complementary techniques, all to be carried out opportunistically.

### 3.3. Dielectric Properties and Conductivity

**Figure 4:**
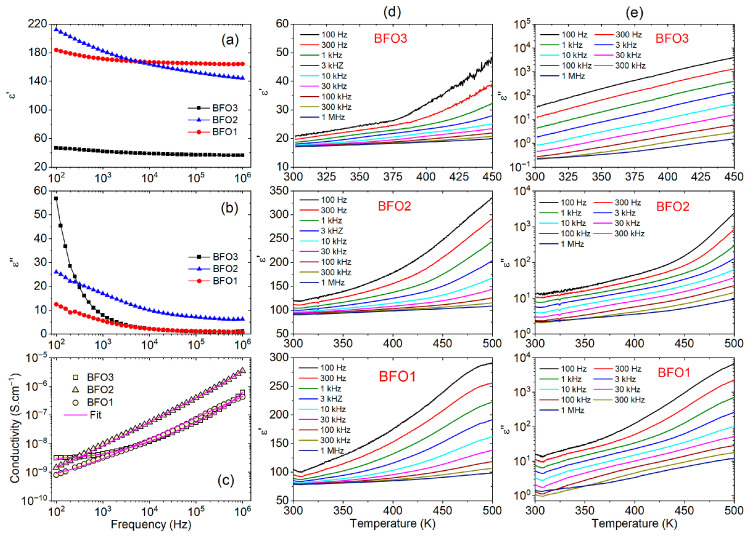
Frequency dependence of (**a**) real ($\varepsilon^{\prime }$ and (**b**) imaginary (*ε*″) dielectric permittivity, and (**c**) ac conductivity (*σ_ac_*) of the studied BiFeO_3_ thin films (BFO1, BFO2, and BFO3) at room temperature. The red lines in (**c**) indicate theoretical fits using the Jonscher model. Temperature dependence of the (**d**) real and (**e**) imaginary dielectric permittivity at different frequencies of the studied BFO1, BFO2, and BFO3 thin films.

Defects such as oxygen vacancies, valence changes from Fe^3+^ to Fe^2+^ ions, and other point defects significantly modify the dielectric and electrical properties of BiFeO3 thin films. The frequency dependence of the real ($\varepsilon^{\prime }$) and imaginary ($\varepsilon^{\prime \prime }$) parts of the dielectric permittivity at room temperature of the studied BiFeO_3_ thin films (BFO1, BFO2, and BFO3) is shown in Figure 4a and Figure 3b. All films show normal dielectric dispersion in both figures, i.e., $\varepsilon^{\prime }$ and $\varepsilon^{\prime \prime }$ decrease with increasing frequency and gradually stabilize above ~ 10^4^ Hz. In Figure 4a, a lower permittivity ($\varepsilon^{\prime }$ = 37 at 100 kHz) almost frequency independent was observed for the BFO3 film, pyrolyzed at 360 °C and crystallized at 640 °C in an O_2_ atmosphere, while higher permittivities ($\varepsilon^{\prime }$ = 153 and 165 at 100 kHz) increasing at low frequencies (~100 Hz) are observed for the BFO1 and BFO2 films, both pyrolyzed at 300 °C and crystallized at 600 °C, in an open atmosphere (in air) and post-annealed in an O_2_ atmosphere, respectively. Although the obtained permittivity values agree with different reports in the literature for BiFeO_3_ thin films prepared from chemical or physical routes, it is important to note that the lower permittivity of the BFO3 film indicates that pyrolysis and crystallization at higher temperature and in an O_2_ atmosphere were effective in minimizing point defects, compared to BFO1 and BFO2 films with higher defects. In addition, post-annealing in an O_2_ atmosphere for the BFO2 film was favorable for the formation of secondary phases and introduced additional defects, allowing mobile charge carriers to accumulate at interfaces or grain boundaries, resulting in significant space charge polarization known as the Maxwell-Wagner-Sillars interfacial polarization [47]. Thus, the higher apparent dielectric permittivity of these last films indicates a space charge polarization effect, which is more pronounced at lower frequencies, as clearly observed in the BFO2 film.

In Figure 4b, BFO3 exhibits the highest dielectric loss $\varepsilon^{\prime \prime }$ (~57) at low frequencies, indicating possibly higher leakage currents. At frequencies above ~10^4^ Hz, BFO2 exhibits a higher $\varepsilon^{\prime \prime }$ (~10), while BFO1 and BFO3 show very similar behavior and much lower losses (~2), with BFO1 having the lowest value, approaching zero at high frequencies (~0.7 at 1 MHz). The decrease in both $\varepsilon^{\prime }(f)$ and $\varepsilon^{\prime \prime }(f)$, with increasing frequency, can be understood by considering carriers in the dielectric material hopping from highly conductive grains to grain boundaries with lower conductivity, from highly conductive grains compared to grain boundaries ($R_{gb}$ > $R_{g}$), in an analogy with the heterogeneous model of the polycrystalline structure given by Koops [48]. This means that increasing the frequency of the applied field decreases the probability that carriers reach the grain boundary. Consequently, the polarization produced by the migratory species decreases as it accumulates at the grain boundaries. Thus, the observed behaviors for $\varepsilon^{\prime }(f)$ and $\varepsilon^{\prime \prime }(f)$ in Figure 4a,b have become comprehensive in the view of this model. As the decrease of $\varepsilon^{\prime \prime }$ reflects the reduced conductive contribution in the sample, lower values of $\varepsilon^{\prime \prime }$ indicate thin films with lower conductivities.

Figure 4c shows the frequency-dependent ac conductivity ($\sigma_{ac}$) of BiFeO_3_ thin films (BFO1, BFO2, and BFO3) in the range 10^2^–10^6^ Hz, plotted on a log–log scale. The ac conductivity can be described by the power-law relation $\sigma \left(\omega \right)=\sigma \left(0\right)+A\omega^{n}$, as proposed by Jonscher [49]. Often used for different materials, $\sigma \left(0\right)=\sigma_{dc}$ in this equation is the frequency-independent conductivity, $A\omega^{n}$ represents the dielectric dispersion, while the exponent n and the coefficient A are parameters dependent on temperature and material. The red lines in Figure 4c are theoretical fits using the Jonscher model. For the BFO3 film, the frequency-independent plateau-like region observed at low frequencies (f < 1 kHz) is attributed to the $\sigma_{dc}$ conductivity, which is expected to be frequency-independent at σ(f→0). The plateau at room temperature and lower frequencies suggests a dc conduction behavior, indicating a single relaxation process in the BFO3 film [50]. On the other hand, no plateaus were observed in the conductivity curves of the BFO1 and BFO2 films within the studied frequency range. From the fittings in Figure 4c, the obtained dc conductivities at room temperature were $\sigma_{dc}$ = 1.2 × 10^−10^, 3.6 × 10^−10^, and 2.7 × 10^−9^ S.cm^−1^ for the BFO1, BFO2, and BFO3 films, respectively. These results are comparable with those reported in the literature for BiFeO_3_ thin films obtained from rf-sputtering ($\sigma_{dc}$~2.8 × 10^−10^ S.cm^−1^ [50]) and for films obtained by chemical solution method ($\sigma_{dc}$~8.0 × 10^−8^ S.cm^−1^), whose differences indicate the influence of the synthesis route.

The temperature dependence of the real ($\varepsilon^{\prime }$) and imaginary ($\varepsilon^{\prime \prime }$) dielectric permittivities of the studied BiFeO_3_ thin films (BFO1, BFO2, and BFO3), measured at different frequencies from 100 Hz to 1 MHz, in the temperature range 300–500 K, are shown in Figure 4d,e, respectively. For the BFO1 and BFO2 films, both $\varepsilon^{\prime }$ and $\varepsilon^{\prime \prime }$ increase gradually with temperature for all frequencies, showing a stronger rise above ~400 K. For these films, the pronounced increase in $\varepsilon^{\prime }$ near 450–500 K suggests space-charge or interfacial polarization contributions, possibly at grain boundaries, since the temperature dependence indicates thermally activated polarization processes. The frequency dispersion in $\varepsilon^{\prime \prime }$ below ~ 10 kHz corroborates the presence of space-charge relaxation arising from inhomogeneous microstructures induced by defects. A similar increase in $\varepsilon^{\prime }$ with temperature is also observed in BFO3 film, with a sharp rise above ~375 K, but the increase in $\varepsilon^{\prime \prime }$ is approximately linear on a log scale, following an Arrhenius-type loss behavior.

### 3.4. Impedance Spectroscopy Analysis

**Figure 5:**
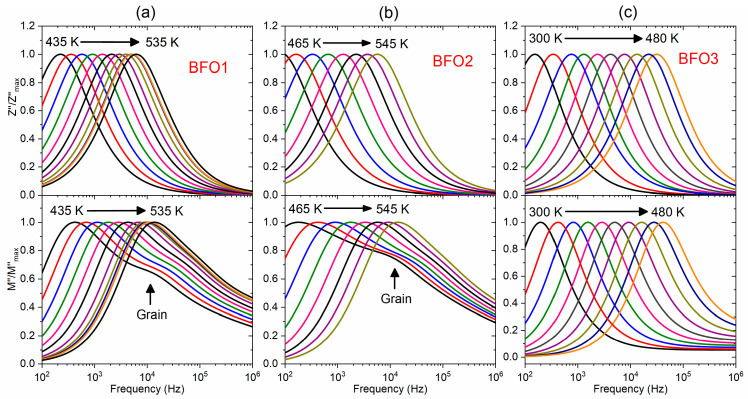
Frequency dependence of the normalized imaginary parts ($Z^{\prime \prime }/Z_{max}^{\prime \prime }$ of impedance and ($M^{\prime \prime }/M_{max}^{\prime \prime }$) electric modulus for (**a**) BFO1, (**b**) BFO2, and (**c**) BFO3 thin films at selected temperature range.

Impedance spectroscopy is a powerful tool for separating the contributions of grains and grain boundaries to the transport properties of materials, providing important insights into the dielectric relaxation mechanisms. Figure 5 shows the frequency dependence of the normalized imaginary parts ($Z^{\prime \prime }/Z_{max}^{\prime \prime }$) of impedance and ($M^{\prime \prime }/M_{max}^{\prime \prime }$) of electric modulus for the studied BiFeO_3_ thin films (BFO1, BFO2, and BFO3) at the selected temperature range. The peaks shifting to high frequencies with increasing temperature confirm a thermally activated dielectric relaxation in these BiFeO_3_ films, so that the hopping mechanism of charge carriers dominates. Increasing the temperature provides more thermal energy to the charge carriers, allowing them to move faster and leading to a decrease in relaxation time and a peak shift towards higher frequencies. Only a single relaxation peak ($Z^{\prime \prime }/Z_{max}^{\prime \prime }$) was observed for BFO1, BFO2, and BFO3 films in Figure 5 (top), while for the electric modulus in Figure 5 (bottom), a single relaxation peak ($M^{\prime \prime }/M_{max}^{\prime \prime }$) was observed for BFO3 film in Figure 5c, but the presence of two peaks were observed for BFO1 and BFO2 films in Figure 5a,b), which can be associated with grain and grain boundary contributions. Point defects, especially oxygen vacancies, increase the number of mobile charge carriers and have a significant effect on the electric modulus ($M^{\prime \prime }$) peak. Since defect-related carriers respond more readily to the alternating electric field, the M″ peak often shifts to higher frequencies, indicating a faster relaxation process. However, if defects are more complex and trap charge carriers, they can shift the spectrum to lower frequencies, reflecting slower relaxation. From Figure 5 (bottom), the peak of the electric modulus ($M^{\prime \prime }$) at 480 K was observed at ~3.7 kHz, 570 Hz, and 38.4 kHz for BFO1, BFO2, and BFO3 films, respectively. It indicates that the relaxation peak shifts to lower frequencies for the BFO1 and BFO2 films, compared to the BFO3 film. The pyrolysis of BFO1 and BFO2 films at a low temperature (300 °C) differs significantly from that of the BFO3 film at a higher temperature (360 °C) in promoting the formation of oxygen vacancies due to incomplete organic removal. This partially explains the high concentration of oxygen vacancies in the BFO1 and BFO2 films, and the low concentration in the BFO3 film, as shown in Figure 3c. Although the post-annealing in an O_2_ atmosphere for the BFO2 film potentially reduces the oxygen vacancies compared to the BFO1 film, crystallized in an open atmosphere at the same temperature, the process is favorable to the crystallization of traces of secondary phases, introducing additional defects that favor the trapping of charge carriers, which leads to the observed shift in the $M^{\prime \prime }$ peak to lower frequencies.

**Figure 6:**
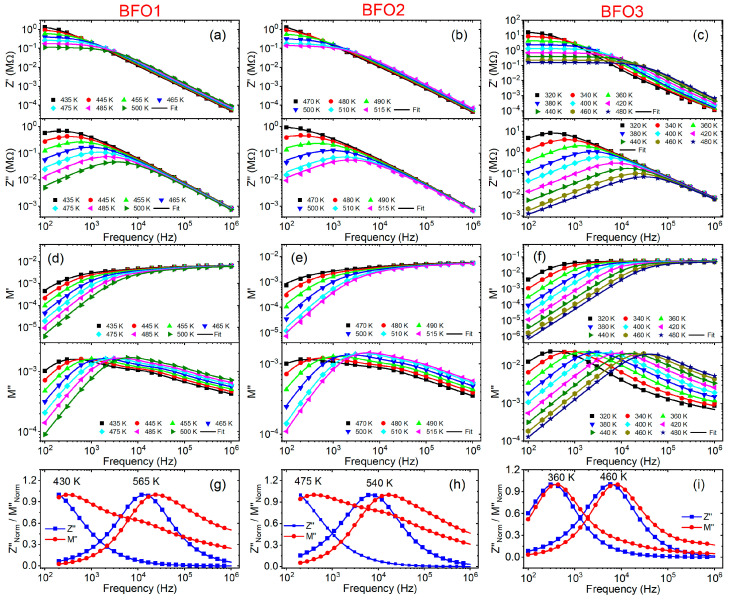
Frequency dependencies of real ($Z^{\prime }$ and imaginary ($Z^{\prime \prime }$) impedances for the (**a**) BFO1, (**b**) BFO2, and (**c**) BFO3 films and real ($M^{\prime }$) and imaginary ($M^{\prime \prime }$) electric modulus for the (**d**) BFO1, (**e**) BFO2, and (**f**) BFO3 films at selected temperatures. Comparison of the $Z^{\prime \prime }$ and $M^{\prime \prime }$ for (**g**) BFO1, (**h**) BFO2, and (**i**) BFO3 films as a function of the frequency at two different temperatures. In all cases, the lines are theoretical fits of Equations (1)–(4).

Figure 6a–c shows the frequency dependence of the real ($Z^{\prime }$) and imaginary ($Z^{\prime \prime }$) parts of the impedance of the BFO1, BFO2, and BFO3 films, at selected temperatures, while the frequency dependence of the real ($M^{\prime }$) and imaginary ($M^{\prime \prime }$) electric modulus is shown in Figure 6d–f. The lines in these figures refer to the theoretical results obtained by using Equations (1)–(4). The plots in these figures are shown on a logarithmic scale to explicitly demonstrate the good agreement between the experimental data and the theoretical fits, as the fit accuracy can be obscured when the impedance data are presented on a linear scale. The good fits of $M^{\prime }$ and $M^{\prime \prime }$, considering the effects of the grain boundary at low frequencies and the grain at high frequencies, confirm the presence of a non-Debye type of relaxation in these samples.

Figure 6g–i compare the frequency dependence of the normalized imaginary impedance ($Z^{\prime \prime }$) and imaginary electric modulus ($M^{\prime \prime }$) at two particular temperatures for the studied BFO1, BFO2, and BFO3 films. These combined plots of impedance ($Z^{\prime \prime }$) and electric modulus ($M^{\prime \prime }$) as a function of frequency are a valuable tool for inferring the conduction mechanism and distinguishing relaxation processes associated with the short-range or long-range motion of charge carriers in ferroelectric thin films [51]. An appreciable mismatch is observed at the $Z_{max}^{\prime \prime }$ and $M_{max}^{\prime \prime }$ of the BFO1 and BFO2 films, while an almost perfect overlap is observed for the BFO3 film. Slight overlapping of the Z″ and M″ peaks confirms that the relaxation process departs from ideal Debye-type behavior and indicates the predominance of grain-boundary response in the BFO3 film within the studied frequency and temperature range, corroborating what was observed in Figure 5c.

For several solid materials, the frequency at which the $Z^{\prime \prime }(f)$ and $M^{\prime \prime }(f)$ peaks occur correspond to the transitions from long-range (delocalized) to short-range (localized) relaxation. The overlapping of the $Z^{\prime \prime }(f)$ and $M^{\prime \prime }(f)$ peaks indicate long-range relaxation, whereas an appreciable mismatch between these peaks is associated with the coexistence of both short and long-range relaxation [52]. In typical dielectric materials, localized relaxation dominates so that the electrical response is more effective in terms of permittivity and dielectric loss. On the other hand, long-range conductivity dominates ionic conductive materials so that the impedance and the electric modulus better reflect the response. For a physical process that exhibits a distribution of relaxation time, the imaginary parts of the impedance and the electric modulus show different relaxation times, such that they are separated $Z^{\prime \prime }\;$ and $M^{\prime \prime }$ peaks characterize short-range relaxation, and overlapped peaks indicate long-range relaxation [52]. Thus, the two partially overlapping relaxation $Z^{\prime \prime }\;$ and $M^{\prime \prime }$ peaks in Figure 6i suggest a predominance of long-range relaxation in the BFO3. In contrast, the observed mismatch between the $Z^{\prime \prime }\;$ and $M^{\prime \prime }$ peaks in Figure 6g,h indicate the coexistence of both short- and long-range relaxation in the BFO1 and BFO2 films, most likely associated with the coexistence of electron/hole hopping and the diffusion of oxygen vacancies, which are most likely introduced by low-temperature pyrolysis during the synthesis of these films.

**Figure 7:**
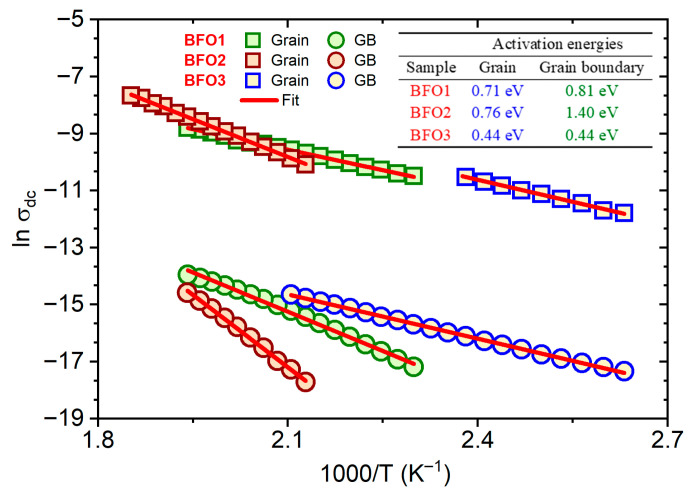
DC conductivity as a function of the reciprocal temperature for the studied BFO1, BFO2, and BFO3 thin films, discriminating the grain and grain boundaries contributions. The results summarized in this figure were obtained from impedance and electric modulus theoretical fits in Figure 6. The inserted table summarizes the grain and grain boundary activation energies obtained from linear fits (red lines).

The activation energies were evaluated from the Arrhenius relations for the dc conductivity $\sigma =\sigma_{0}exp(-E/k_{B}T)$, where $\sigma_{0}$ is a pre-exponential factor, $k_{B}$ is the Boltzmann constant, and E is the activation energy. From the $R_{g}$ and $R_{gb}$ values obtained from theoretical fits of the impedance and the electric modulus in Figure 6, the dc conductivities ($\sigma_{dc}$) associated with the grain and grain boundary were obtained, considering the relation $\sigma_{dc}=d{\left(Ra\right)}^{-1}$, where d (film thickness) and a (electrode area) are geometrical parameters. Thus, Figure 7 shows the reciprocal temperature dependence $\sigma_{dc}$ for both the grain and the grain boundary, whose respective activation energies were evaluated from linear fits in the figure. The inset table in Figure 7 summarizes the obtained activation energies for grains and grain boundaries. The grain and grain boundary activation energies were 0.71 eV and 0.81 eV for the BFO1 film, and 0.76 eV and 1.40 eV for the BFO2 film. Both films were pyrolyzed at 300 °C, crystallized at 600 °C in an open atmosphere (in air), and post-annealed in an O2 atmosphere, respectively. On the other hand, for the BFO3 film, pyrolyzed and crystallized at a higher temperature and in an O_2_ atmosphere, the activation energies for the grain and the grain boundary were the same and equal to 0.44 eV. The different activation energies indicate that distinct mechanisms are responsible for electrical conduction in the studied films, indicative of defects introduced during synthesis.

In the literature, different values of activation energies have been reported for BiFeO_3_ thin films. For films prepared by rf-sputtering, two distinct conduction activation energies of 0.37 eV and 0.73 eV were reported [50], attributed respectively to the first ionization of oxygen vacancies and to electron hopping for conduction at low temperatures, and to the thermal excitation of carriers from the second ionization of oxygen vacancies. For La-Ti-co-doped BiFeO_3_ films, the activation energies of 0.46 and 0.47 eV for the grain and grain boundary, respectively, were associated with the first ionization of oxygen vacancies [50]. Furthermore, studies on the fatigue process of polycrystalline BiFeO_3_ thin films revealed that defect evolution during polarization switching reduces the activation energy from 0.9 eV to 0.2 eV [53]. Independent of defect type, there is consensus that oxygen vacancies play an essential role in the mechanism responsible for dielectric relaxation and electric conduction in BiFeO_3_ [50]. In this scenario, the grain and grain boundary activation energies for the BFO1 (0.71 eV and 0.81 eV) and BFO2 films (0.76 and 1.40 eV) indicate a conduction mechanism associated with the second ionization of oxygen vacancies in these films. In contrast, the first ionization of oxygen vacancies governs the conduction mechanism in both grains and grain boundaries of the BFO3 film (0.44 eV), a consequence of reduced defect concentration in this sample.

### 3.5. Leakage Current and Photovoltaic Properties

**Figure 8:**
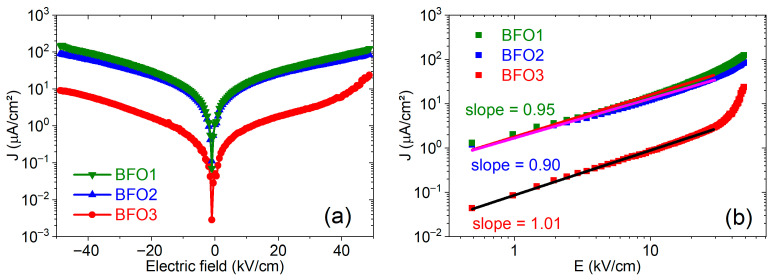
(**a**) J–E leakage current curves and (**b**) J–E curves in log–log plots for the positive branch in (**a**) of the studied BFO1, BFO2, and BFO3 thin films at room temperature. Straight lines are linear fits.

Figure 8a shows the typical leakage current density electric field (J−E) characteristics of the studied BiFeO_3_ films in the dark condition, measured at room temperature for both negative and positive biases in the electric field range of ±50 kV/cm. In this figure, the J−E  curves show similar shapes with the positive and negative branches symmetrical for the BFO1 and BFO2 films, pyrolyzed at 300 °C, crystallized at 600 °C in an open atmosphere (in air), and post-annealed in an O_2_ atmosphere. In contrast, for the BFO3 film, pyrolyzed and crystallized (in O_2_ atmosphere) at higher temperatures, the positive and negative branches are slightly asymmetrical, such that its leakage current density (23 μA/cm^2^) at 50 kV/cm is one order of magnitude lower than that of the BFO1 (123 μA/cm^2^), and 3.6 times smaller than the leakage current density for the BFO2 (84 μA/cm^2^) film, which present a similar behavior compared to BFO1.

A leakage current in BiFeO3 thin films has often been attributed mainly to oxygen vacancies [19], as several studies have shown that reducing their concentration minimizes leakage current in ferroelectric thin films. As demonstrated by the RIR values from XPS results, the BFO1 film shows the highest concentration of oxygen vacancies among the samples studied, the BFO2 film shows an intermediate concentration, and the BFO3 film shows the lowest concentration. The observed leakage current density follows the concentration of oxygen vacancies in the studied films, suggesting a correlation. However, the mechanisms of leakage current can be more intricate and may correlate with defects associated with the Bi/Fe and Fe^3+^/Fe^2+^ ratios rather than with the oxygen vacancy concentration. Deviation changes the Fe ions’ valence from Fe^3+^ to Fe^2+^, leading to highly conductive samples, while the equation $null\to {2V}_{Bi}^{\prime \prime \prime }+{3V}_{O}^{\cdot \cdot }+{Bi}_{2}O_{3}$ describes the origin of bismuth vacancies and the consequent Bi_2_O_3_ formation [52]. Bi vacancies introduce three holes in the system, and the presence of Fe^2+^ and oxygen vacancies with different valence states justifies the presence of free electrons. In the BiFeO_3_ system, deep localized oxygen vacancy traps the holes, and the charge neutrality condition determines the equivalence or not between the free electrons and holes [15]. Thus, it is plausible that free electrons and holes compensate for the charge, mutually remaining in a competition between Bi and oxygen vacancies. This scenario shows that the synthesis process is fundamentally important to control defects during the preparation of BiFeO_3_ thin films. According to EDS analyses, Bi/Fe ≈ 1 for all studied films within experimental accuracy, whereas XPS analyses indicate Fe^3+^/Fe^2+^ ≈ 2 for all films, indicating the coexistence of Fe^2+^ and Fe^3+^ ions, with the predominance of Fe^3+^ ions. Although the presence of a small amount of secondary phases in films BFO2 and BFO3 should act as significant sources and mediators for various defects, the results indicate that oxygen vacancies are the main factor contributing to the leakage current in the studied films.

The analysis of conduction mechanisms offers additional insights into the conductivity. Among different mechanisms, the ohmic is given by the linear relation J=σE, where σ is the electrical conductivity and generally dominates in the regime of low electric fields. The SCLC mechanism is provided by the relation $J=[(9\varepsilon_{0}\varepsilon_{r}\mu )/(8d)]E^{2}$, where  μ is the charge mobility, $\varepsilon_{0}$ the vacuum permittivity, $\varepsilon_{r}$ the low-frequency relative dielectric permittivity and d the sample thickness [54,55]. Figure 8b shows log(J) versus log(E) plots of the studied BFO films for positive bias. The linear fits to the J(E) curves at electric fields below 30 kV/cm yield slopes ~1 for all films, as shown in Figure 8b, confirming ohmic conduction at low electric fields and excluding the SCLC mechanism for these films within the studied electric field range.

**Figure 9:**
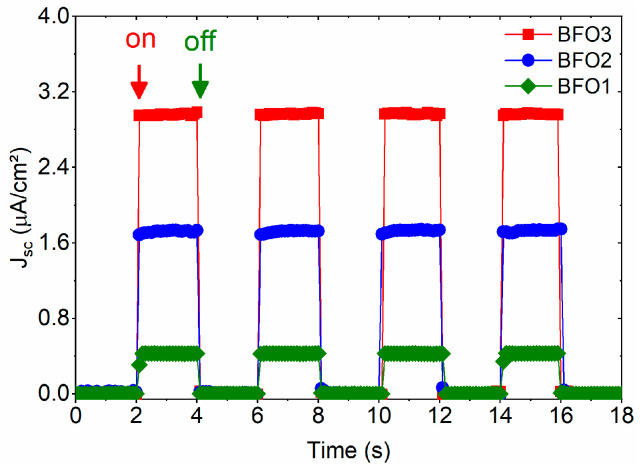
Photocurrent density (JSC) of the studied BFO1, BFO2, and BFO3 thin films measured at zero bias under monochromatic green light (λ = 532 nm), under the regime “light on” and “light off” illumination.

The measured photocurrent densities (JSC) of the studied BiFeO_3_ films at short-circuit conditions are shown in Figure 9 under light-on and light-off illumination, alternated for 2 s and repeated 4 times. Under illumination, all studied BFO films show a clear and distinct photovoltaic response. The most significant photocurrent density of the BFO3 film ($J_{SC}\;$~2.97 μA/cm^−2^) is about 6.9 times higher than the lowest value observed for the BFO1 film ($J_{SC}\;$~0.43 μA/cm^−2^), and about 1.7 times higher than the photocurrent density of the BFO2 film ($J_{SC}\;$~1.72 μA/cm^−2^). The highest photocurrent density of the BFO3 film is in the same order as those reported for BiFeO_3_ films prepared by the chemical solution deposition method on Pt/Ti/SiO_2_/Si (100) substrates ($J_{SC}\;$~7.50 μA/cm^−2^) [56] or on FTO substrate ($J_{SC}\;$~10.96 μA/cm^−2^) [19], but one magnitude order smaller than that observed for films prepared by the sol–gel method on Pt/Ti/SiO_2_/Si substrate ($J_{SC}\;$~20.3 μA/cm^−2^) [57]. These differences are comprehensible in this qualitative comparison, since the photocurrent responses in the present work were obtained under green light illumination (λ = 532 nm) while those reported were under near-ultraviolet (λ = 405 nm) illumination or solar simulator.

The respective maximum and minimum photocurrent densities for the BFO3 and BFO1 films in Figure 9, along with their lower and higher leakage currents in Figure 8a, confirm that a high oxygen vacancy concentration is mainly responsible for the photocurrent degradation in the studied BiFeO_3_ thin films. The influence of defects on the leakage current and conduction mechanisms of BiFeO_3_ thin films has been widely studied [8,9], as well as the relationship between their leakage current and photovoltaic response [19]. In these cases, the oxygen vacancies acted as trap centers that greatly influenced the recombination of photogenerated carriers. Comparing the BFO1 (crystallized in air) and BFO2 films (post-annealed in O_2_), both pyrolyzed (300 °C) and crystallized (600 °C) at lower temperatures, it was observed that post-annealing in O2 reduces the oxygen vacancies, thereby favoring the crystallization of traces of secondary phases. As traces of secondary phases are also present in the BFO3 film, pyrolyzed and crystallized at higher temperatures in an O2 atmosphere, it is clear that defects associated with these phases are dominant over the reduction of oxygen vacancies. Although the photocurrent response depends on a combination of defects, the oxygen vacancies still dominate. The results indicate that increasing the pyrolysis temperature can be more effective for reducing oxygen vacancies during synthesis by the chemical method, thereby improving photovoltaic performance.

### 3.6. Magnetic Properties

**Figure 10:**
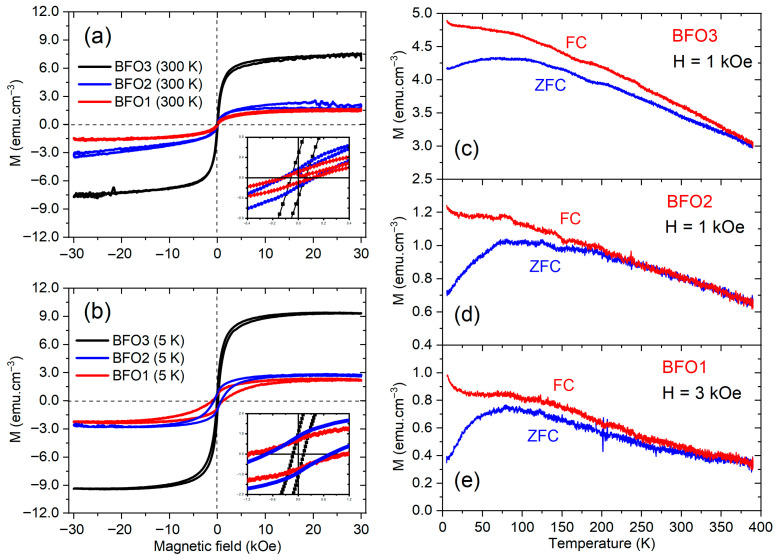
(**a**) Magnetic hysteresis loops (M−H taken at 300 K temperature and (**b**) at 5 K of the studied BFO1, BFO2, and BFO3 thin films. ZFC and FC magnetization curves as a function of temperature for the (**c**) BFO3 (at *H* = 1 kOe), (**d**) BFO2 (at *H* = 1 kOe), and (**e**) BFO1 (at *H* = 3 kOe) films.

The magnetic properties of the studied films were also characterized. Figure 10 shows the magnetization hysteresis loops (M−H) of BFO1, BFO2, and BFO3 thin films, measured at 300 K and 5 K in Figure 10a,b, respectively, with an applied magnetic field within ± 30 kOe. The diamagnetic contribution was removed from all magnetic hysteresis loops by subtracting the high-field linear slope from the raw data in these figures. The well-saturated M−H hysteresis at 300 K and 5 K temperatures suggests the ferromagnetic nature of BiFeO_3_ thin films. It is evident in Figure 10a,b that the saturation magnetization is higher for the BFO3 and minimum for the BFO1 film, and intermediate for the BFO2 film, at high temperature (300 K) as well as at low temperature (5 K). From the magnified M−H curves inserted in Figure 10a,b, it is observed that the remanent magnetization ($M_{r}$) for BFO3 film ($M_{r}$ = 0.4 emu/cm^3^) is almost four times higher than both BFO1 and BFO2 films ($M_{r}$ = 0.1 emu/cm^3^) at 300 K, while all films showed essentially the same ($M_{r}$ = 0.8 emu/cm^3^) at 5 K. On the other hand, the coercive field is almost the same ($H_{c}$ ≈ 813 Oe) for BFO1 and BFO2 films and smaller ($H_{c}$ = 128 Oe) for the BFO3 film at 300 K, showing the same behavior at 5 K with higher coercive fields.

) taken at 300 K temperature and (**b**) at 5 K of the studied BFO1, BFO2, and BFO3 thin films. ZFC and FC magnetization curves as a function of temperature for the (**c**) BFO3 (at H = 1 kOe), (**d**) BFO2 (at H = 1 kOe), and (**e**) BFO1 (at H = 3 kOe) films.

Figure 10c,d,e shows the magnetization curves of the BFO3, BFO2, and BFO1 films, respectively, as a function of temperature in the zero-field cooling (ZFC) and field cooling (FC) regimes at H = 1 and 3 kOe applied fields, as indicated. While the ZFC and FC curves show the same shape for all studied films, the magnetization magnitude is similar for the BFO1 and BFO2 films, differing from the higher magnetization of the BFO3 film. The magnetization of the BFO3 film is greater than that of the BFO2 and BFO1 films at any given temperature. In the ZFC regime, the magnetization of the BFO3 film is 5 times greater than that of the BFO2 film and 8 times greater than that of the BFO1 film at 300 K, and at 5 K it is 6 times greater than that of the BFO2 film and 12 times greater than that of the BFO1 film. The magnetization in the FC regime decreases monotonically with increasing temperature for all films, whereas the curves in the ZFC regime exhibit a maximum magnetization at the blocking temperature ($T_{B}$). This temperature is generally attributed to a spin-glass-like transition or to a typical blocking process for superparamagnetic spin moments, in which the magnetic moments of independent ferromagnetic regions become “blocked” below this temperature; in other words, they are unable to reorient themselves with temperature changes in the ZFC process. Above this temperature, the thermal energy is sufficient to allow the moments to randomly fluctuate [58]. Although the blocking temperature is not an intrinsic characteristic of the BiFeO3 thin films, $T_{B}$ is an important characteristic, especially for applications in spintronics, which depends on synthesis conditions, such as film thickness, composition, and strain [59]. In the present study, based on the curves shown in Figure 10, $T_{B}$ presents essentially the same value around 79 K for all the studied films.

Despite the blocking temperature in BiFeO_3_ thin films being a dynamic parameter that often depends on processing conditions or point defects, in the present work, this temperature was apparently unaffected, showing essentially the same value across all films prepared under different conditions. On the other hand, the magnetization magnitude, remanent magnetization, and coercive field showed clear dependence on point defects, such as oxygen vacancies, which are strongly influenced by processing conditions. The high oxygen vacancy concentration and low crystallinity in BFO1 and BFO2 films decrease their magnetization and remanent magnetization, while increasing their coercive field, compared to the BFO3 film, at low (5 K) and room temperature (300 K).

## 4. Conclusions

In summary, BiFeO_3_ thin films were synthesized by chemical solution deposition under different conditions to investigate the impact of oxygen vacancies on their physical properties and to identify the ideal conditions to minimize their formation during synthesis. High oxygen vacancies were observed in films prepared at low pyrolysis temperature (300 °C) and crystallized in air, while the oxygen vacancies decreased considerably, increasing the pyrolysis temperature (360 °C) in the film crystallized in an O_2_ atmosphere. Furthermore, for the film crystallized in air, subsequent prolonged heat annealing in an O2 atmosphere partially removed the oxygen vacancies, but the results demonstrated that, once established during synthesis, there are practically no alternatives to completely correct them through post-crystallization thermal annealing under the experimental conditions. As reported in several studies, the physical properties of the samples studied were strongly affected by the presence of oxygen vacancies. The dielectric loss, dielectric dispersion, dc conductivity, grain activation energy, and leakage current increased in the presence of oxygen vacancies, degrading the photovoltaic and magnetic properties of the studied films. The study reinforces the importance of controlling oxygen vacancies during synthesis by establishing the ideal conditions for obtaining high-quality thin films.

## Figures and Tables

**Table 1:** Summary of the conditions used for the preparation of the studied BiFeO_3_ thin films.

Sample	Pyrolysis	Crystallization	Post Annealing (In O_2_)
BFO1	300 °C/60 min	600 °C/40 min (in air)	No
BFO2	300 °C/60 min	600 °C/40 min (in air)	600 °C/5 h
BFO3	360 °C/10 min	640 °C/40 min (in O_2_)	No

**Table 2:** Atomic percentages of the elements in the studied BiFeO_3_ thin films obtained from the statistical analysis of the EDS data.

Sample	Bi (atm%)	Fe (atm%)	O (atm%)	Bi/Fe	O/Bi	O/Fe
BFO1	17.0 ± 0.4	15.7 ± 0.4	67.3 ± 0.8	1.08 ± 0.08	4.0 ± 0.2	4.3 ± 0.2
BFO2	17.5 ± 0.5	16.5 ± 0.6	66 ± 1	1.06 ± 0.07	3.8 ± 0.2	4.0 ± 0.2
BFO3	21.5 ± 0.3	21.6 ± 0.3	57.0 ± 0.6	1.00 ± 0.02	2.7 ± 0.4	2.6 ± 0.1

**Table 3:** Summary of structural parameters obtained from the Rietveld refinement of the XRD patterns of the studied BiFeO_3_ thin films. Despite rather high R_wp_ and R_p_ values, the $\chi^{2}$ values are a good indication of reliable fits, as evidenced by the visual inspection of data in Figure 2a. Reasons for suboptimal R_wp_ and R_p_ values may include a small interaction volume due to data collected in thin films instead of bulk crystals, inherent incoherence and strains at the substrate/film interface, and multiphase refinements due to the presence of Pt signals, among others.

Sample	Lattice Parameters	Atomic Positions	x	y	z	R-Factors
BFO1	*a* = 5.569 (9) Å	Bi^3+^	0.0000	0.0000	0.0000	R_wp_ = 27.93%
	*c* = 13.833 (8) Å	Fe^3+^	0.0000	0.0000	0.2159	R_p_ = 24.09%
	*V* = 371.68 Å^3^	O^2−^	0.4188	0.0223	0.9668	$\chi^{2}$ = 1.017
BFO2	*a* = 5.571 (5) Å	Bi^3+^	0.0000	0.0000	0.0000	R_wp_ = 26.96%
	*c* = 13.849 (7) Å	Fe^3+^	0.0000	0.0000	0.2115	R_p_ = 22.96%
	*V* = 372.08 Å^3^	O^2−^	0.4450	0.0180	0.9503	$\chi^{2}$ = 1.003
BFO3	*a* = 5.571 (5) Å	Bi^3+^	0.0000	0.0000	0.0000	R_wp_ = 27.30%
	*c* = 13.847 (4) Å	Fe^3+^	0.0000	0.0000	0.2243	R_p_ = 20.01%
	*V* = 372.26 Å^3^	O^2−^	0.4450	0.0180	0.9503	$\chi^{2}$ = 1.937

**Table 4:** Assignment and Raman modes obtained in the present work in comparison with results from the literature for BiFeO_3_ single crystals and epitaxial BiFeO_3_ thin films.

Symmetry	BFO1 (cm^−1^)	BFO2 (cm^−1^)	BFO3 (cm^−1^)	Crystal (cm^−1^) [35]	Film (cm^−1^) [36]
A_1_	144	141	142	140	135
A_1_	177	175	177	173	172
A_1_	216	215	217	220	218
E	261	261	261	265	266
E	282	282	283	279	277
E	349	349	350	350	350
E	372	372	372	371	365
A_1_	430	430	425	-	-
E	474	474	473	471	465
E	487	487	489	-	-
E	529	528	528	550	548

## Data Availability

The original contributions presented in this study are included in the article. Further inquiries can be directed to the corresponding authors. The raw data supporting the conclusions of this article will be made available by the authors on request.
